# Supplementary material for: Small Extracellular Vesicles Derived from Lipopolysaccharide-Treated Stem Cells from the Apical Papilla Modulate Macrophage Phenotypes and Inflammatory Interactions in Pulpal and Periodontal Tissues
Source: Int J Mol Sci. 2024 Dec 31;26(1):297. doi: 10.3390/ijms26010297 (PMC11719611; doi:10.3390/ijms26010297)
Supplement: Supplementary file 1 [file ijms-26-00297-s001.zip › ijms-3383707-supplementary.pdf]

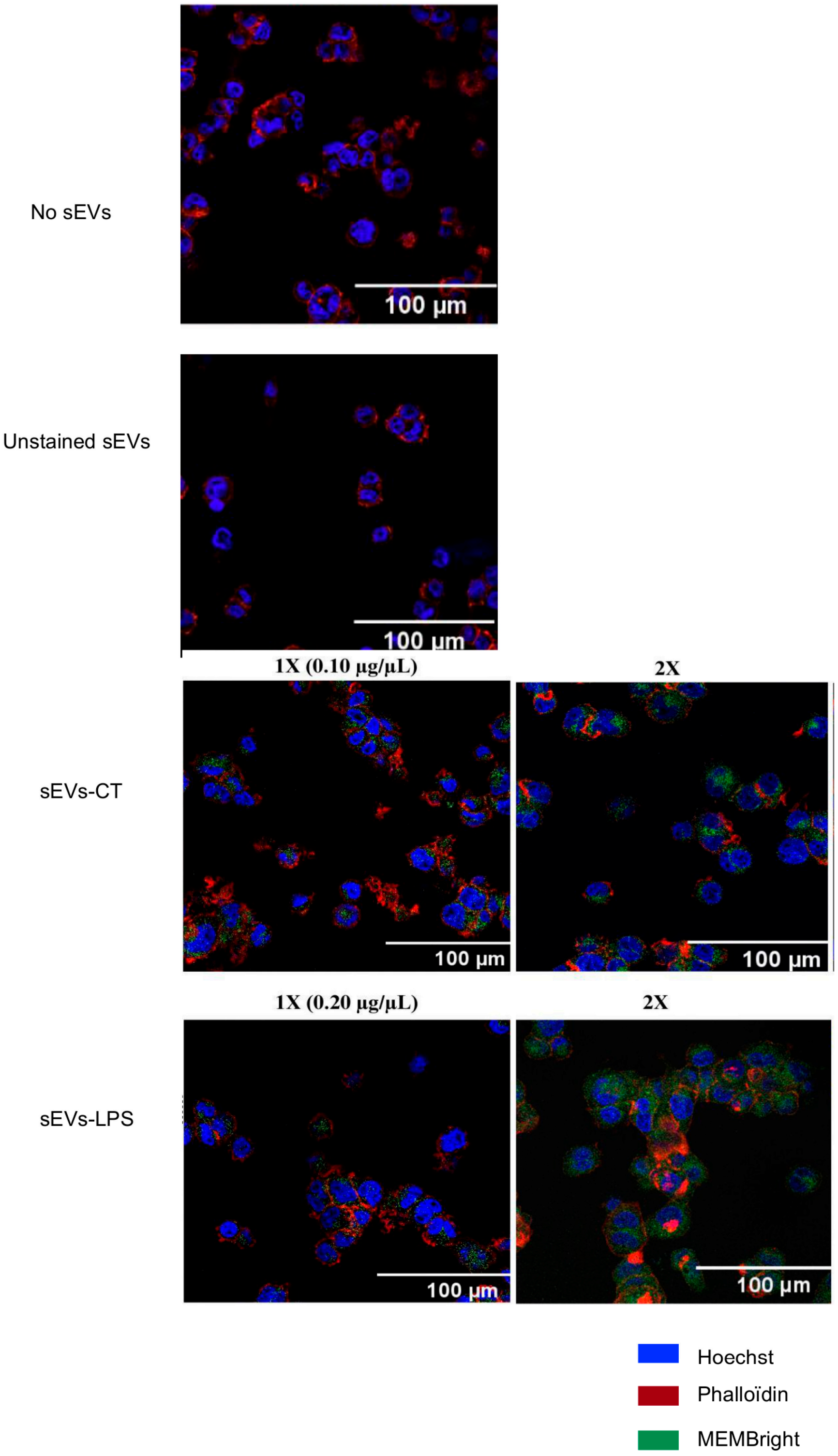

Figure S1. Confocal microscopy images of THP-1 cells under various conditions. The images are composed of several Z-projection series. Conditions tested include MEM Bright – PBS, unlabeled VE NS 1X, sEVs-CT 1X, sEVs-CT 2X, sEVs-LPS 1X, and sEVs-LPS 2X. Small extracellular vesicles (sEVs) were labelled with the green marker MEMBright488. Actin filaments within the cells were stained with red Phalloidin, and cell nuclei were labelled in blue with Hoechst.

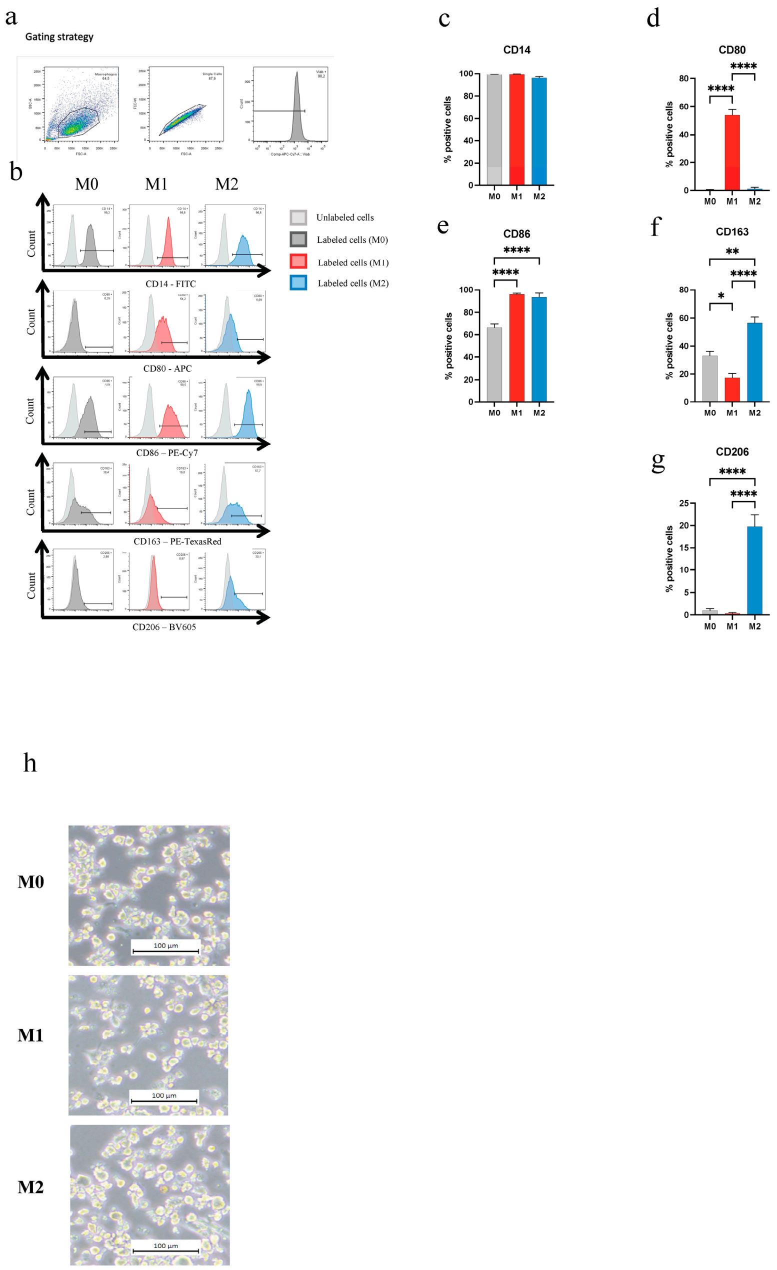

Figure S2. Characterization of M1 and M2 macrophages derived from PMA-treated THP-1 Cells. (**a**) Gating strategy and (**b**)representative flow cytometry histograms of cell surface M1 and M2 macrophages markers with quantitative comparison of percentages of cell surface M1 and M2 macrophages markers analyzed by FACS CD14 (**c**), CD80 (**d**), CD86 (**e**), CD163 (**f**), CD206 (**g**). P-values derived from one-way ANOVA followed by Tukey's multiple comparison tests are * $P < 0.05$, ** $P < 0.01$, and **** $P < 0.0001$. (**h**) Light microscopic observation of cell morphology of M0, M1 and M2.
